# Validation of Self-Reported Cancer Diagnosis by Cognitive Status in the Health and Retirement Study

**DOI:** 10.1093/geroni/igab046.628

**Published:** 2021-12-17

**Authors:** Megan Mullins, Jasdeep Kler, Marissa Eastman, Mohammed Kabeto, Lauren Wallner, Lindsay Kobayashi

**Affiliations:** University of Michigan, Ann Arbor, Michigan, United States

## Abstract

Exploring the relationship between cognition and cancer is increasingly important as the number of older adults in the US grows. The Health and Retirement Study (HRS) has longitudinal data on cognitive status and self-reported cancer diagnoses, but these self-reports have not been validated. Using HRS linked to Medicare Fee for Service (FFS) claims (1998-2016), we evaluated the validity of self-reported cancer diagnoses (excluding non-melanoma skin) against Medicare claims by respondent cognitive status. We included 8,280 Medicare-eligible HRS participants aged ≥67 with at least 90% FFS coverage. Cognitive status was ascertained from the HRS interview following the date of cancer diagnosis (or reference claim date) using the Langa-Weir method and was classified as normal, cognitive impairment no dementia (CIND), or dementia. We calculated the sensitivity, specificity, and Cohen's kappa for first incident malignant cancer diagnosis by cognitive status group. The majority (76.4%) of participants scored as cognitively normal, 9.6% had CIND, 14.0% had dementia and, overall, 1,478 had an incident cancer diagnosis. Among participants with normal cognition, sensitivity of self-reported cancer diagnosis was 70.2% and specificity was 99.8% (kappa=0.79). Among participants with CIND, sensitivity was 56.7% and specificity was 99.8% (kappa=0.66). Among participants with dementia, sensitivity was 53.0% and specificity was 99.6% (kappa=0.64). Results indicate poor validity of self-reported cancer diagnoses for older adults with CIND or dementia. These findings suggest researchers interested in cancer and cognition should use the HRS-Medicare linkage to ascertain cancer diagnosis from claims, and they highlight the importance of cognitive status in research among older adults.

